# Global burden of anemia and cause among children under five years 1990–2019: findings from the global burden of disease study 2019

**DOI:** 10.3389/fnut.2024.1474664

**Published:** 2024-10-15

**Authors:** Yujuan Liu, Weifang Ren, Shuying Wang, Minmin Xiang, Shunxian Zhang, Feng Zhang

**Affiliations:** ^1^Department of Pharmacy, Jinshan Hospital Affiliated to Fudan University, Shanghai, China; ^2^Longhua Hospital, Shanghai University of Traditional Chinese Medicine, Shanghai, China; ^3^Department of Emergency, Jinshan Hospital Affiliated to Fudan University, Shanghai, China

**Keywords:** anemia, iron deficiency, children, etiology, disease burden

## Abstract

**Background:**

Anemia represents a significant global health issue affecting numerous children and women, characterized by diminished hemoglobin levels that may impede cognitive and developmental progress. Although commonly attributed to iron deficiency, the etiology of anemia in this demographic is multifaceted, encompassing nutritional, genetic, and infectious contributors. Nonetheless, there is a lack of high-quality data on anemia prevalence and causes analysis among children under 5 years. The aim of this study was to provide a comprehensive global assessment of the burden of anemia and its causes among children under 5 years, using data from the Global Burden of Disease Study 2019.

**Methods:**

This investigation utilized data from the Global Burden of Disease Study (GBD) 2019 to assess the prevalence and years lived with disability (YLD) attributable to anemia in children under five from 1990 to 2019. Analyses were conducted to delineate age-specific YLD, prevalence rates, and etiological factors, with stratification by gender and Socio-Demographic Index (SDI).

**Results:**

In 2019, anemia imposed a substantial global burden on children under five, with a reported YLD of 1,252.88 (95% UI: 831.62–1,831.34) per 100,000 population. The prevalence of moderate anemia was highest at 985.46 (95% UI: 646.24–1,450.49) per 100,000, surpassing both severe anemia at 197.82 (95% UI: 132.53–277.80) per 100,000 and mild anemia at 69.59 (95% UI: 24.62–152.53) per 100,000. Globally, the total prevalence was 39,517.75 (95% UI: 38,784.81 - 40,239.62) cases per 100,000 population. Notably, disparities were evident between genders, with males demonstrating higher YLD and prevalence rates than females. Iron deficiency emerged as the leading cause globally, with significant contributions from hemoglobinopathies and other nutritional deficiencies. Regions with a low Socio-Demographic Index, particularly sub-Saharan Africa and South Asia, exhibited the most pronounced burdens. Despite a declining trend over three decades, persistent regional and gender-based disparities highlight the necessity for continuous and focused public health interventions.

**Conclusion:**

The burden of anemia among children under five continues to be considerable, marked by stark regional and socioeconomic disparities. These findings underscore the urgent need for advanced nutritional and healthcare strategies tailored to alleviate anemia in this vulnerable population, with a particular emphasis on regions exhibiting low SDIs. The sustained prevalence of high anemia rates in these areas underscores the imperative for persistent, localized intervention efforts.

## Introduction

Anemia represents a significant global health challenge, particularly among the most vulnerable populations, including children under the age of five (1). A reduction in either the quantity or functionality of red blood cells and hemoglobin characterizes anemia. This condition impairs the blood’s capacity to transport oxygen efficiently. As a result, individuals with anemia often experience symptoms such as fatigue, weakness, and shortness of breath (2, 3). The condition’s etiology is multifaceted, encompassing nutritional deficiencies, genetic disorders, infectious diseases, and more (4). This complexity necessitates a comprehensive understanding of anemia’s various causes and their specific impacts on different demographic groups, especially young children (5–7). Their heightened vulnerability to anemia’s adverse effects underscores the importance of early detection, prevention, and intervention strategies to safeguard their health and development.

The epidemiological landscape of anemia is diverse and influenced by an interplay of genetic, environmental, and socio-economic factors. Globally, anemia affects approximately one-fourth of the population, with pronounced disparities observed across regions, ages, and genders (8–10). The prevalence is notably higher in developing countries, where factors such as malnutrition, inadequate healthcare infrastructure, and a higher burden of infectious diseases like malaria and HIV/AIDS exacerbate the risk of anemia (11, 12). Children under five in these regions are particularly affected, facing increased morbidity and mortality risks (8). Anemia’s impact extends beyond immediate health concerns, contributing to long-term developmental delays, impaired cognitive function (11, 13), and diminished educational performance (14, 15), thereby perpetuating cycles of poverty and disadvantage.

Despite significant advances in understanding anemia’s global burden (7), a gap remains in current, comprehensive data that delineates the complex causes and consequences of anemia in children under 5 years. The GBD data, while extensive, lacks focus on this specific age group, and detailed findings are yet to be made public. This study leverages the most recent 2019 GBD data to examine the point prevalence, YLDs, and attributable causes of anemia in children under 5 years globally from 1990 to 2019. Through this analysis, we aim to enhance the global understanding of anemia’s impact on younger populations, guiding future research and informing prevention, diagnosis, and management strategies to mitigate the health burden of anemia among children and adolescents.

## Methods

### Overview of the global burden of disease study 2019

The GBD Study 2019 offers a comprehensive analysis of health challenges worldwide, covering 369 diseases and injuries along with 87 risk factors from 1990 to 2019, across 204 countries and territories (16). Utilizing robust methodologies, previously detailed in extensive publications, this study highlights trends in epidemiological patterns. The GBD 2019’s extensive dataset, accessible via the Global Health Data Exchange (GHDX) and the interactive GBD Compare platform, serves as an invaluable resource for delineating the evolving landscape of global health challenges, including both fatal and non-fatal health outcomes.

### Definitions and classification of anemia

Anemia is defined in this study by age-adjusted reduced hemoglobin levels, following specific criteria for classification. This analysis, spanning the years 1990 to 2019 across 204 nations, calculates the distribution of hemoglobin concentrations, anemia prevalence, and YLDs due to anemia across 37 identified etiologies, covering all genders and 25 age categories. The gradation of anemia into mild, moderate, and severe categories is based on hemoglobin concentration thresholds that vary by age, gender, and pregnancy status, in alignment with World Health Organization standards and adapted for specific demographic nuances.

### Methodological approach to assessing anemia prevalence

The assessment of anemia prevalence in children under five utilized demographic health surveys, scholarly articles, and governmental reports. Data were aligned with specific geographic, age, gender, and pregnancy demographics, following GBD study protocols, including adjustments for elevation based on the WHO formula. The method did not alter hemoglobin sample collection or analysis techniques. Spatiotemporal Gaussian process regression and linear mixed-effects models estimated hemoglobin levels and anemia prevalence, applying inverse weighting for model accuracy. Discrepancies were smoothed across various dimensions, enhancing the precision of uncertainty quantification in the final results.

### Assessment of YLDs

The YLDs calculation for anemia in children under five was conducted by correlating the number of anemia cases at each severity level with severity-specific disability weights. These weights represent the degree of health impairment on a scale from 0 (no impairment) to 1 (death), allowing for a nuanced understanding of anemia’s impact on early childhood development.

### Causal attribution of anemia

Our analysis identified several causes of anemia in children under five, employing a mutually exclusive and collectively exhaustive approach to assign each anemia case to one of the 37 defined causes. This involved utilizing distributions of hemoglobin concentration, overall anemia prevalence by severity, disease prevalence or incidence, and cause-specific hemoglobin shifts derived from extensive research. This detailed methodology facilitates a comprehensive understanding of anemia’s etiology in young children, which is essential for targeted health interventions.

### Analysis of anemia trends in relation to socio-economic progress

We explored the relationship between anemia prevalence in children under five and socio-economic development, analyzing how anemia’s prevalence and impact on YLDs correlate with the SDI across different regions. Employing meta-regression models, we investigated the potentially non-linear relationships between anemia burden and SDI, identifying countries where anemia prevalence significantly deviates from expectations based on socio-economic status. This multifaceted analysis elucidates the influence of socio-economic factors on anemia prevalence and disability in young children, informing health policy and resource distribution.

## Results

### Global level

Anemia’s impact on the global population of children under five in 2019 was profound, with the total YLDs amounting to 1,252.88 (95% UI: 831.62–1,831.34) per 100,000 population. A nuanced breakdown by severity reveals the differential impact of the condition: mild anemia contributed to a YLDs rate of 69.59 (95% UI: 24.62–152.53) per 100,000 population, moderate anemia presented a considerably higher YLDs rate of 985.46 (95% UI: 646.24–1,450.49) per 100,000 population, and severe anemia resulted in a YLDs rate of 197.82 (95% UI: 132.53–277.80) per 100,000 population (Supplementary Table S1).

Furthermore, the prevalence of anemia in 2019 among this demographic elucidates the extent of its reach. The overall prevalence was reported at a rate of 39,517.75 (95% UI: 38,784.81 - 40,239.62) per 100,000 population. Disaggregating this data by severity, mild anemia exhibited a prevalence rate of 18,880.01 (95% UI: 18,527.70 - 19,193.64) per 100,000, moderate anemia impacted 19,281.86 (95% UI: 18,822.14 - 19,795.88) per 100,000, and severe anemia was less common but still significant, with a prevalence rate of 1,355.87 (95% UI: 1,272.73 - 1,444.12) per 100,000 (Supplementary Table S1).

### Regional level

The analysis reveals stark regional disparities in childhood anemia, correlated with the SDI. Low SDI regions face the most severe burden, with a YLDs rate of 2310.58 per 100,000 population (95%UI: 1,522.92–3,346.29). This rate decreases in low-middle SDI regions to 1,487.04 (95% UI: 975.95–2,185.34), and further to 1,252.88 (95% UI: 831.62–1,831.34) globally. Higher SDI regions experience significantly lower rates, with middle, high-middle, and high SDI regions reporting YLDs of 725.25 (95% UI: 475.04–1,072.18), 448.08 (95% UI: 286.17–679.03), and 159.53 (95% UI: 97.23–251.78) respectively. In terms of prevalence, 67.43% of children under five in low SDI regions suffer from anemia, contrasting with only 14.40% in high SDI areas. Western and Central Sub-Saharan Africa record the highest rates, emphasizing extreme regional disparities in the impact of anemia.

### National level

**Figure 1:**
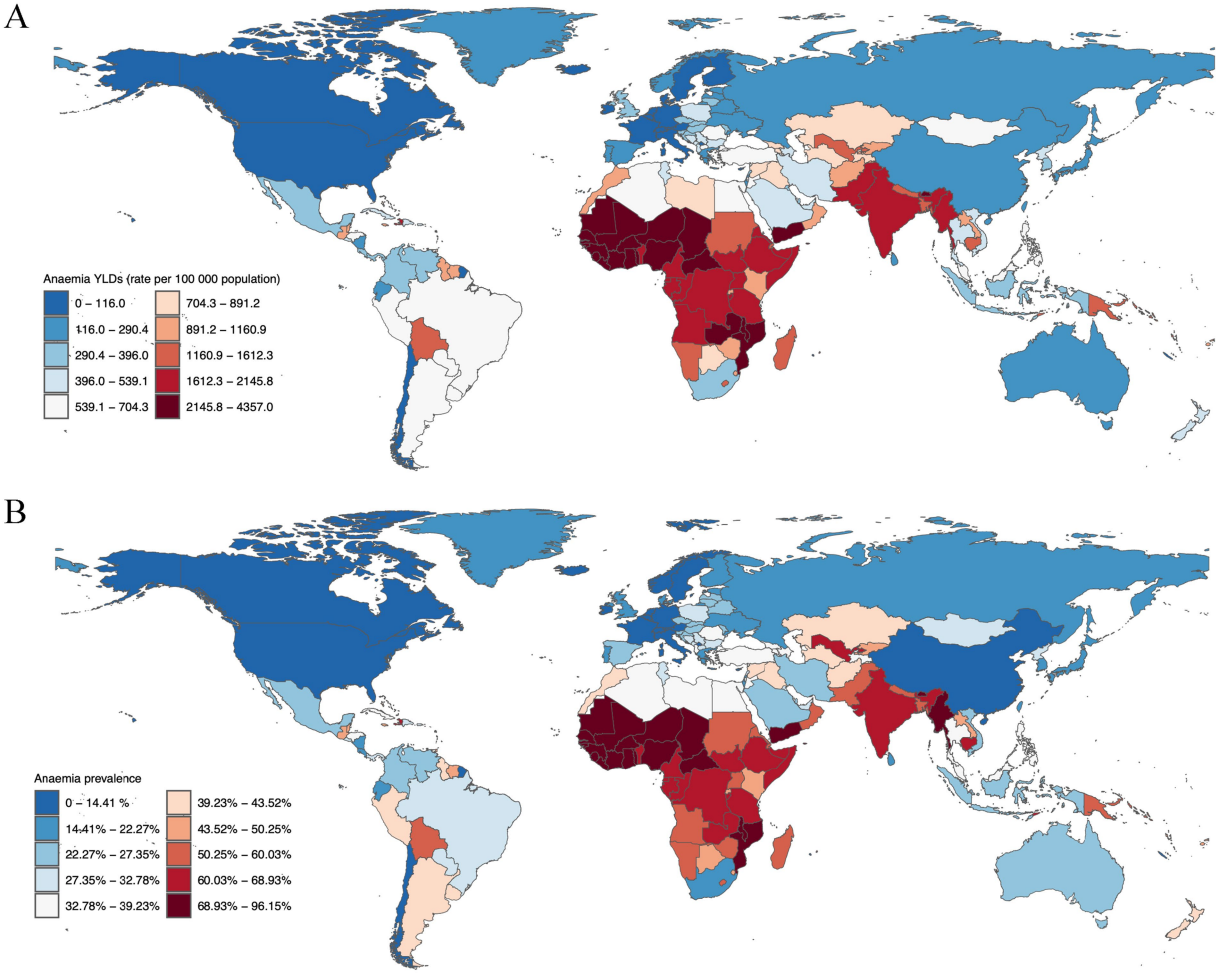
Anaemia burden in children under five, 2019. **(A)** Anaemia YLDs (rate per 100,000 population) for children under 5 years old, separated by gender, 2019. **(B)** Anaemia prevalence for children under 5 years old, separated by gender, 2019.

Next, we elucidate the national disparities in the burden and prevalence of anemia among children under the age of 5 years in 2019. Notably, Yemen emerged as the country with the highest YLDs rate for anemia among children under five, registering 4,356.99 YLDs per 100,000 (95%UI: 2,894.25 - 6,249.51). This was closely followed by Burkina Faso with a rate of 4,060.77 YLDs per 100,000 (95% UI: 2,743.77 - 5,856.50), and Bhutan, which recorded a rate of 3,543.98 YLDs per 100,000 (95% UI: 2,266.97 - 5,124.99). Other countries with significant YLDs rates included Mali (3,458.41 YLDs, 95% UI: 2,288.55 - 4,958.01), Sierra Leone (3,085.13 YLDs, 95% UI: 2,019.33 - 4,577.64), and the Central African Republic (2,682.17 YLDs, 95% UI: 1,644.53 - 4,000.34), underscoring the acute burden of anemia in these nations (Figure 1A).

The prevalence of anemia among children under five further illustrates the severe impact of this condition. Bhutan reported the highest prevalence at 96.15% (95% UI: 89.49–100%), closely followed by Yemen with a prevalence of 92.61% (95% UI: 87.99–97.10%), and Burkina Faso at 88.95% (95% UI: 84.75–92.69%). Mali (83.86, 95% UI: 78.97–88.13%), Sierra Leone (81.71, 95% UI: 77.08–86.08%), and the Central African Republic (80.82, 95% UI: 72.86–87.86%) also exhibited high prevalence percentages, indicating a widespread impact across these regions (Figure 1B).

### Temporal trends and gender disparities

Globally, YLDs attributable to anemia have generally decreased over the past three decades for both genders across all age groups, with males consistently showing higher rates than females. For neonates (0–6 days), male YLDs decreased from 576.08 (95% UI: 369.47 to 859.27) to 541.04 (95% UI: 346.39 to 816.84), while female YLDs went from 336.29 (95% UI: 207.35 to 538.78) to 323.95 (95% UI: 197.22 to 517.40). In the 7–27 days age group, male YLDs saw a temporary rise from 2,557.11 (95% UI: 1,667.66 to 3,760.10) to 2,648.77 (95% UI: 1,745.83 to 3,843.95), contrasting with a decrease in females from 2,426.25 to 2,288.63. Both genders exhibited a downward trend in the 28–364 days category, with male YLDs reducing from 1,645.17 to 1,511.28, and female YLDs from 1,600.82 to 1,413.55. Children aged 1–4 years also saw declines, with male YLDs dropping from 1,556.87 to 1,225.95, and females from 1,455.92 (95% UI: 957.66 to 2,104.46) to 1,153.24 (95% UI: 755.59 to 1,688.78).

**Figure 2:**
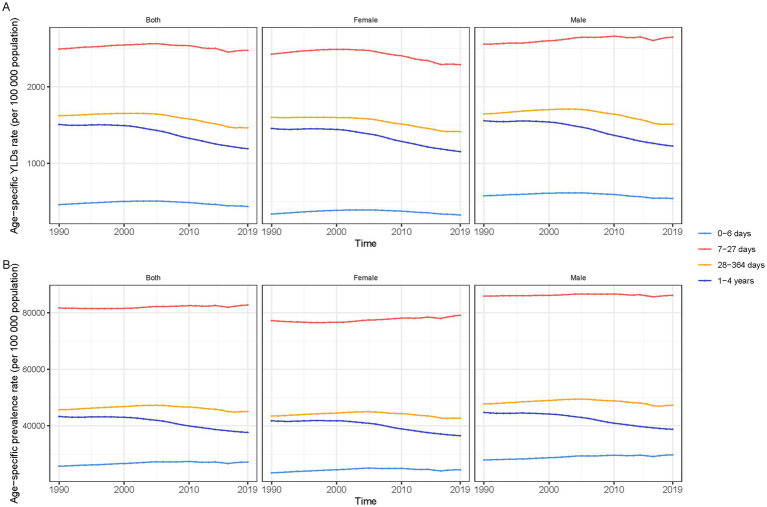
Trends in anaemia burden among children under five by age group and gender, 1990–2019, globally. **(A)** Anaemia YLDs (rate per 100,000 population). **(B)** Anaemia prevalence.

Prevalence trends varied by age. Newborns (0–6 days) saw an increase in anemia rates, with male rates rising from 27,938.12 per 100,000 to 29,744.13 per 100,000, and female rates from 23,356.06 per 100,000 to 24,406.13 per 100,000. Infants aged 7–27 days experienced slight increases in prevalence, while those in the 28–364 days group saw minor declines. The most notable decrease occurred in children aged 1–4 years, with male prevalence dropping from 44,744.73 per 100,000 to 38,777.18 per 100,000, and female rates from 41,780.57 per 100,000 to 36,499.94 per 100,000 (Figure 2).

### Causal attribution of anemia

Our findings reveal that dietary iron deficiency emerges as the leading global cause of anemia-related YLDs, followed in significance by hemoglobinopathies and hemolytic anemias. Other notable causes include neglected tropical diseases, malaria, vitamin A deficiency, and unspecified infectious diseases. The highest incidence rates were observed in tropical Latin America, South Asia, and both western and eastern sub-Saharan Africa, indicating geographical variations in anemia etiology.

**Figure 3:**
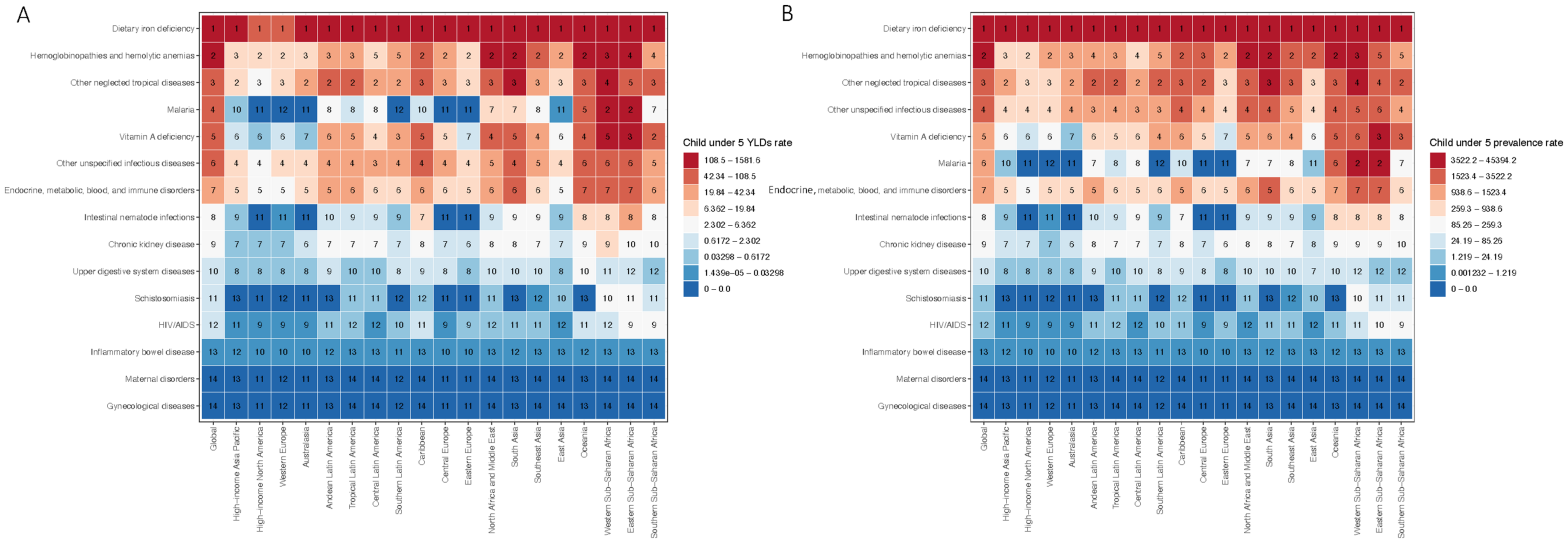
Global and regional rankings of anemia-related burden in children under five. **(A)** Global and regional rankings of anemia-related YLDs by cause in children under five, 2019. **(B)** Global and regional rankings of anemia with prevalence by cause in children under five, 2019.

Significant disparities in the predominant causes of anemia were noted globally and within specific regions (Figure 3A). While dietary iron deficiency was identified as the primary cause of anemia-related YLDs on a global scale and across most regions, the African regions exhibited a higher relative burden from malaria, neglected tropical diseases, and hemoglobinopathies/hemolytic disorders.

A detailed heat map analysis of the prevalence rates attributable to 15 causes of anemia among children under five further corroborates the variability in anemia etiology (Figure 3B). Consistent with the YLDs data, dietary iron deficiency ranked as the foremost contributor to the child anemia burden globally, succeeded by hemoglobinopathies and hemolytic anemias. Other significant causes encompassed neglected tropical diseases, malaria, vitamin A deficiency, and unspecified infectious diseases, with the highest prevalence rates recorded in tropical Latin America, South Asia, and sub-Saharan Africa.

### The burden of age-specific leading causes of anemia

**Figure 4:**
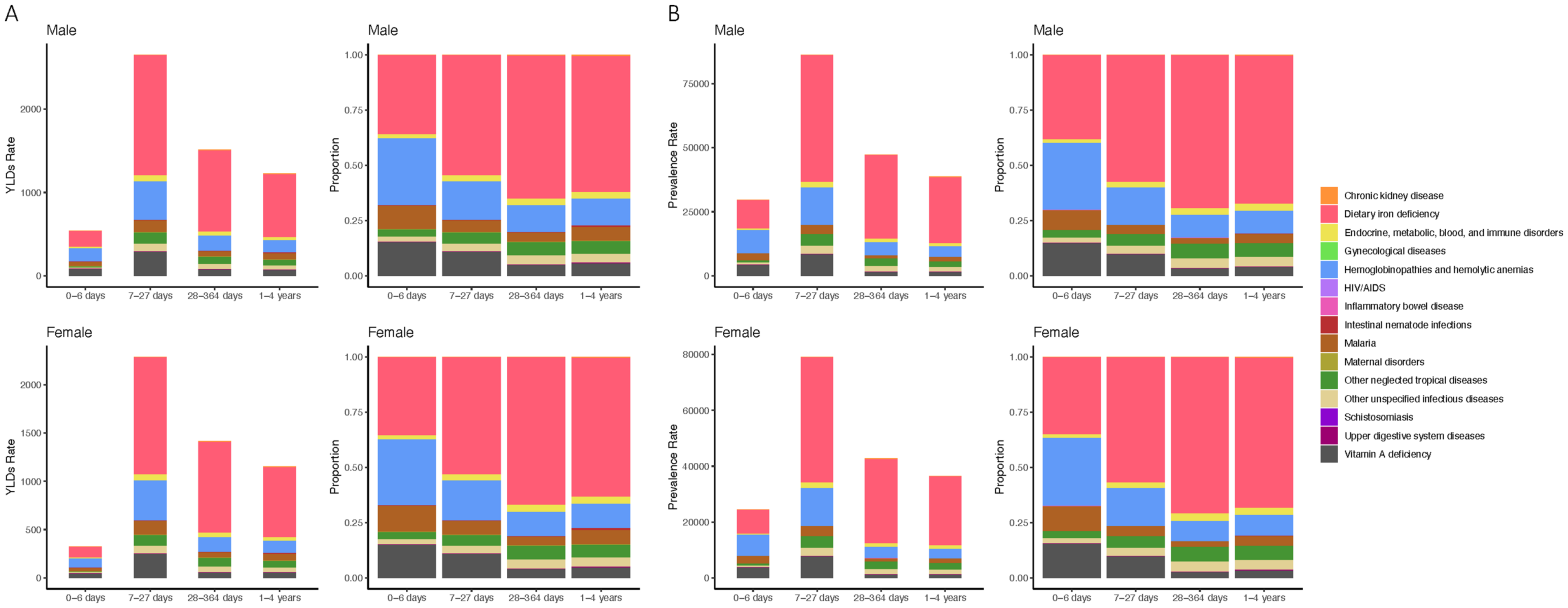
Anemia etiological contributors, prevalence, and YLDs rates by age and gender. **(A)** YLDs rate and proportional distribution by cause in male and female children under five across four age categories. **(B)** Prevalence rate and proportional distribution by cause in male and female children under five across four age categories.

In neonates, hemoglobinopathies are the primary contributors to anemia-related YLDs, accounting for 25% in males and 30% in females, followed by dietary iron deficiency which impacts 30% of males and 36% of females. Vitamin A deficiency affects 13% of males and 15% of females, with malaria contributing to around 9%. During the early infancy stage (7–27 days), dietary iron deficiency dominates, representing 35% of male and 37% of female anemia YLDs, with hemoglobinopathies causing around 12%. Vitamin A deficiency and malaria also contribute, albeit less significantly. For infants aged 28 to 364 days, dietary iron deficiency remains the largest factor, influencing 49% of male and 50% of female YLDs, while hemoglobinopathies affect about 9%. Children aged 1 to 4 years see dietary iron deficiency causing 47% of male and 48% of female anemia YLDs, with hemoglobinopathies contributing to around 6% (Figure 4A).

Prevalence patterns echo these findings, with dietary iron deficiency being most prevalent in neonates, affecting 28% of males and 32% of females, and similarly high figures persist across all age groups. Hemoglobinopathies and vitamin A deficiency remain consistent causes across different ages, while malaria’s impact is relatively lower but persistent (Figure 4B). This comprehensive assessment highlights the varied etiological landscape of anemia, dominated by nutritional deficiencies and genetic disorders across different developmental stages.

### Socio-economic development and its impact on childhood anemia

**Figure 5:**
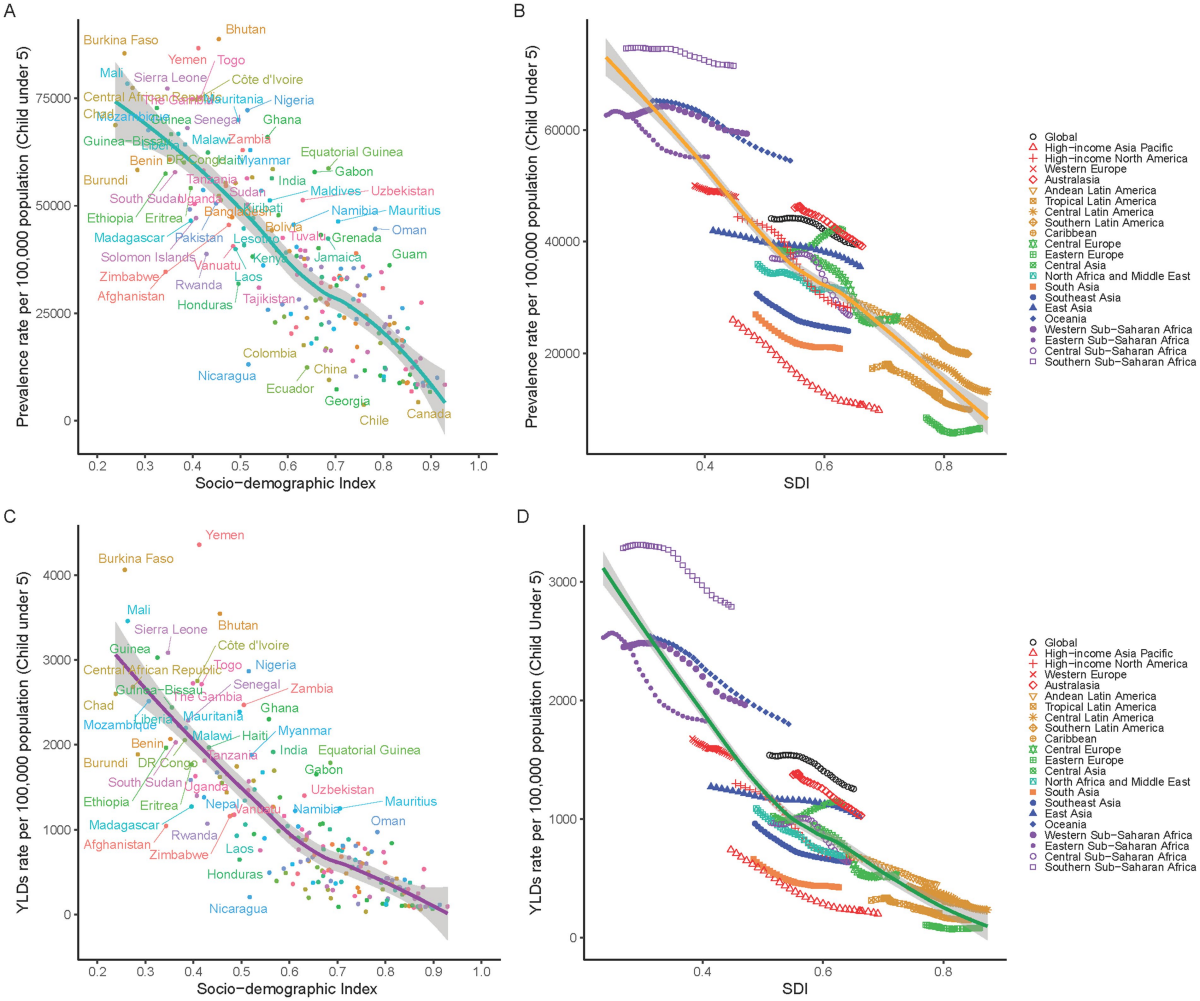
Correlation between SDI and Anemia Burden in children under5 by national and 21 GBD regions. **(A)** Anemia prevalence in children under five at the national level in relation to SDI in 2019. **(B)** Trends in anemia prevalence in children under five across 21 GBD regions, correlated with SDI from 1990 to 2019. **(C)** YLDs rates for anemia in children under five at the national level in relation to SDI in 2019. **(D)** Trends in YLD rates for anemia in children under five across 21 GBD regions, correlated with SDI from 1990 to 2019.

Our study reveals a strong inverse relationship between socio-economic development, measured by the SDI, and childhood anemia rates. Regions with lower SDI values, particularly below 0.5 in sub-Saharan Africa and South Asia, experience high anemia YLDs over 2000 per 100,000 population (Figure 5A). In contrast, more developed regions with SDI values above 0.7, such as North America, Europe, and high-income areas in Asia and Latin America, show significantly lower YLDs under 1000 per 100,000 (Figure 5B). The decline in anemia rates correlates with socio-economic improvements, especially noted in East and Southeast Asia from 1990 to 2019. However, Oceania, with a moderate SDI of 0.6, contradicts this trend, suggesting other factors may influence anemia rates. A similar pattern is observed in anemia prevalence, with high rates in low SDI regions and marked reductions in areas with higher SDIs (Figures 5C,D). Overall, the global data confirms that socio-economic advancement plays a crucial role in reducing the impact of childhood anemia.

## Discussion

This study, leveraging data from the Global Burden of Disease Study 2019, furnishes a comprehensive global overview of anemia in children under 5 years, a paramount public health issue. Despite a global decline in anemia disability rates among adults from 1990 to 2019, the burden of anemia in children under five remains pronounced, signifying the persistent public health challenge anemia poses to this vulnerable demographic.

In 2019, the age-standardized YLDs attributable to anemia in children under five was calculated at 1,252.88 per 100,000. Notably, moderate anemia emerged as the most prevalent form, with a rate of 985.46 per 100,000. Nevertheless, the persistently high YLDs rates among children under five underscore the significant adverse effects of anemia on early developmental stages, potentially resulting in long-term cognitive and physical development impairments, thereby affecting future health and productivity. The observed global reduction in anemia disability rates reflects the impact of effective public health interventions and advancements in nutritional standards (7). This includes fortifying foods with essential nutrients, ensuring access to quality healthcare, and educating communities about the importance of nutrition and regular health check-ups (17).

The disparity in anemia burden between regions with low and high SDI scores in 2019 is particularly striking, with a higher prevalence of anemia reported in low SDI areas (67.43%) compared to high SDI areas (14.40%). The significant contribution of countries like Yemen, the Central African Republic, and Bhutan to the YLDs and prevalence of anemia in children under five underscores the regional disparities in the burden of anemia. Similarly, high YLDs rates in Sierra Leone, Mali, and Burkina Faso point to regional health challenges and resource constraints. These disparities highlight a correlation between socio-economic development, as measured by the SDI, and anemia rates in children, illustrating how economic and social factors profoundly influence public health. Improved living standards, better healthcare access, and enhanced nutrition, often byproducts of higher SDI, contribute to lower anemia rates. Regions with high SDI such as North America, Australasia, Europe, parts of Latin America, and the Asia-Pacific demonstrate the benefits of socio-economic development, exhibiting lower rates of anemia-related YLDs. These areas generally benefit from access to diverse, nutritious foods, comprehensive healthcare systems, and effective public health policies, all of which help to mitigate the burden of anemia (7, 17). These facts indicate the significant impact of socio-economic factors on health and suggests that interventions need to be tailored to the specific needs and constraints of each region. This correlation emphasizes the importance of socio-economic investments in improving living standards, healthcare access, and nutrition to alleviate the global burden of anemia.

Typically, anemia is more common in females, especially during reproductive years, due to menstrual blood loss and the increased iron demands of pregnancy (18–21). Our results reveal a higher anemia disability rate in male children under 5 years. Biological factors such as differences in iron metabolism or genetic predispositions might explain the higher anemia rates in males (22). Males and females have different patterns of iron metabolism, which may contribute to the gender disparity in anemia rates. There could be developmental differences between male and female children in this age group that affect their vulnerability to anemia. For instance, rapid growth phases, differences in gut microbiota, or varying rates of nutrient absorption might influence anemia risk (23). This unexpected trend in early childhood suggests potential biological differences in iron metabolism or genetic predispositions contributing to the increased vulnerability of male children to anemia. These findings suggest the need for gender-sensitive approaches in public health policies addressing childhood anemia. This includes tailored nutritional programs, equitable healthcare access, and awareness campaigns that cater to the specific needs of both male and female children.

For neonates aged 0–6 days, hemoglobinopathies, rather than iron deficiency anemia, are the primary contributors to anemia-related YLDs. Hemoglobinopathies are genetic disorders that affect the structure or production of hemoglobin and are notably significant in the first days of life (24). Conditions such as thalassemia and sickle cell disease often appear shortly after birth, imposing a greater disease burden in this age group than iron-deficiency anemia, which typically manifests later (25). Due to the genetic basis of hemoglobinopathies, prenatal screening and genetic counseling are vital. These services are particularly beneficial for parents with known risk factors or those who carry genes associated with hemoglobinopathies, enabling them to make informed decisions regarding their child’s health (26, 27).

The primary role of dietary iron deficiency in childhood anemia highlights the critical importance of nutrition in public health (7, 28). This emphasizes the need for global initiatives aimed at enhancing dietary quality, particularly with respect to iron consumption, which is essential for both preventing and treating anemia in children (29). Although iron deficiency is a principal cause, the considerable influence of hemoglobinopathies and hemolytic anemias also demands attention. In regions such as Africa, infectious diseases like malaria notably contribute to the anemia burden (12). This suggests that, in addition to nutritional interventions, disease control is a vital component of anemia management in these areas. Comprehensive health policies are therefore necessary to address the multifaceted causes of anemia. Such policies should include food fortification with iron, the expansion of maternal and child health programs, and improved healthcare access (30–32). Specifically in regions like Africa, where certain diseases exacerbate anemia, efforts should focus not only on improving nutrition but also on managing diseases that significantly contribute to anemia, including strategies to reduce the prevalence of malaria and other neglected tropical diseases (11, 33).

### Limitations

This study underscores the multifaceted nature of anemia in children under five. Nonetheless, the current study is subject to certain limitations. Although the GBD study provides a comprehensive dataset, it predominantly depends on extant public health records and research, which may be incomplete or less accurate in specific regions. Consequently, estimates of the anemia burden in particular areas or among distinct demographic groups might not be entirely precise. Moreover, the inherent focus of this epidemiological methodology on quantitative metrics, such as incidence rates and YLDs, may result in the underestimation of the qualitative impact of anemia on individual and community well-being. This limitation could obscure significant socio-economic and psychological dimensions of anemia, thereby restricting the scope of the analysis and its implications for public health interventions.

## Conclusion

This analysis delineates the global burden of anemia in children under five as a complex issue influenced by genetic, nutritional, socio-economic, and region-specific factors. A targeted, multi-faceted strategy is essential, incorporating early detection, nutritional supplementation, improved healthcare access, and regionally tailored interventions. Such approaches are crucial for alleviating the anemia burden and fostering optimal developmental outcomes in this vulnerable demographic, highlighting the necessity of prioritizing childhood anemia in global public health agendas.

## Data Availability

The original contributions presented in the study are included in the article/Supplementary material, further inquiries can be directed to the corresponding authors.
